# The STAR trial: can quality of life benefit offset any survival detriment?

**DOI:** 10.1186/1745-6215-12-S1-A33

**Published:** 2011-12-13

**Authors:** Fiona Collinson, Janet Brown, Christopher McCabe, Julia Brown, Sandy Tubeuf, Barbara Potrata, Jenny Hewison, Ines Rombach, Peter Selby, Catherine Olivier, Helen Howard, Walter Gregory

**Affiliations:** 1 University of Leeds, Leeds, UK

## 

The STAR trial is a UK randomized phase II/III study of first-line sunitinib in locally advanced/metastatic clear cell renal carcinoma (mRCC). It compares the utilization of a sunitinib conventional continuation strategy (CCS) with an experimental sunitinib drug-free interval strategy (DFIS).

Sunitinib is approved for the first-line treatment of mRCC and convention dictates that it is continued until disease progression or unacceptable toxicity. Duration of chemotherapy is frequently determined by cumulative toxicity, but this is not necessarily the case for targeted therapies, and the default has previously been to continue these treatments for longer. A DFIS has the potential advantage of improved quality of life (QoL) and cost-effectiveness, due to longer time-periods off-treatment.

The STAR trial is unique in determining whether QoL benefits from a DFIS can offset any potential detriment in overall survival (OS). Endpoints will assess both survival and QoL separately, but will also assess averaged QALY (quality of life year).

Temporarily stopping a treatment, which is working, is recognized to be potentially challenging for patients, and a qualitative substudy will be performed alongside the phase II trial, to explore patient feelings regarding trial entry and stopping treatment. Results from this will inform recruitment strategies in phase III.

The multi-stage design maximizes resource efficacy by facilitating a seamless transition between the phase II and III parts, assuming attainment of the phase II endpoints (recruitment rate and time to strategy failure). It also enables the QALY data from the initial phase II part of the trial to be used to inform and verify the powering of the overall phase III trial, as minimal data was available during initial trial design to power on a QALY outcome.

Other novel outcome measures (time to strategy failure and summative progression free interval) have also been required, due to the intermittent nature of the DFIS reducing the utility of the standard progression free survival endpoint. With increasing use of targeted therapies and interest in DFIS due to potential QoL and cost effectiveness benefits, these novel endpoints will be increasing required in future clinical trial designs.

The STAR trial will be an exemplar trial in the evaluation of optimal treatment strategies for targeted therapies in other diseases. We will discuss issues relating to the trial design and the methodology relating to the novel endpoints in this study, as well as comment on the implications for future trial design.

